# Protective Effect of Flavonoids from Mulberry Leaf on AAPH-Induced Oxidative Damage in Sheep Erythrocytes

**DOI:** 10.3390/molecules27217625

**Published:** 2022-11-07

**Authors:** Qinhua Zheng, Weijian Tan, Xiaolin Feng, Kexin Feng, Wenting Zhong, Caiyu Liao, Yuntong Liu, Shangjian Li, Wenzhong Hu

**Affiliations:** 1 School of Pharmacy and Food Science, Zhuhai College of Science and Technology, Zhuhai 519041, China; 2 College of Life Science, Jilin University, Changchun 130012, China

**Keywords:** mulberry leaf, flavonoid extraction, erythrocyte hemolysis model, antioxidant activity, AAPH

## Abstract

To evaluate the antioxidant activity of flavonoids extracted from Chinese herb mulberry leaves (ML), flavonoids from mulberry leaves (FML) were extracted and purified by using ultrasonic-assisted enzymatic extraction and D101 macroporous resin. Using LC-MS/MS-Liquid Chromatography with tandem mass spectrometry analysis, hesperidin, rutoside, hyperoside, cyanidin-3-o-glucoside, myricitrin, cyanidin, and quercetin were identified, and NMR and UV were consistent with the verification of IR flavonoid characteristics. The antioxidant activity of FML has also been evaluated as well as the protective effect on 2,2 0-azobis (2-amidinopropane) dihydrochloride (AAPH)-induced oxidative stress. The results showed that FML exhibited powerful antioxidant activity. Moreover, FML showed dose-dependent protection against AAPH-induced sheep erythrocytes’ oxidative hemolysis. In the enzymatic antioxidant system, pretreatment with high FML maintained the balance of SOD, CAT, and GSH-Px; in the non-enzymatic antioxidant system, the content of MDA can be effectively reduced after FML treatment. This study provides a research basis for the development of natural products from mulberry leaves.

## 1. Introduction

Mulberry (*Moru alba* L.) is a perennial woody plant and an important crop, native to central and northern China and mainly distributed in Asia [1]. Mulberry leaves (ML) are often used as feed for silkworms and as one of the homologous Chinese traditional medicines. Numerous studies have shown that the range of biologically active compounds in ML includes 1-DNJ, polysaccharides, flavonoids, polyphenols, vitamins, amino acids, etc. [2,3,4,5,6,7,8]. Recently, however, the flavonoids from mulberry leaves (FML) have attracted more attention and research from scholars because of their various biological activities such as anti-inflammatory, hypoglycemic, hypolipidemic, anti-obesity, anti-aging, and antioxidant activities [2,4,6,8,9,10]. In China, the annual production of ML can reach 20 tons per hectare; however, the utilization rate of ML resources is only 1–3% [11] and there is a serious surplus of ML production relative to the demand for sericulture. Due to the complex composition of ML, it is particularly important to select a suitable extraction method.

Flavonoids as a general term for a class of naturally occurring low molecular weight compounds derived from 2-phenyl chromogenic ketones as a backbone are regarded as the most important polyphenolic compounds, which are ubiquitously found in plants, fruits, and vegetables [12,13].

ML contains a large number of flavonoids such as quercetin, rutinoside, and rutin [14,15]. Kim et al. isolated a number of flavonoids from mulberry leaves such as purpurin and quercetin [16]. Kayo et al. isolated the flavonoid rutinoside from the alcoholic extract of ML [17]. Flavonoids in mulberry leaves (FML) are natural antioxidants with the physiological activity of scavenging free radicals in the human body. It has been proved that FML have hypolipidemic [18], anti-inflammatory [1,19], anti-obesity [20], anti-aging, and antioxidant [20,21] activities.

Recent studies have shown that oxidative stress is involved in the development and progression of various diseases such as cardiovascular diseases, tumors, and inflammation, and is one of the pathogenic mechanisms of such diseases [22,23,24]. Free radicals induce oxidative stress, and antioxidants can scavenge excessive free radicals produced in the body, due to which they have been a hot topic of research. FML are highly effective natural antioxidants that can not only effectively scavenge excess free radicals in the body, but also block the free radical chain reaction, which has both preventive and chain-breaking effects. It has been reported in the literature that FML have free radical scavenging effects [19] and can increase the activity of antioxidant enzymes in normal cells. 

AAPH oxidative inducer is commonly used in erythrocyte oxidative hemolysis models. It has been widely used in in vitro cellular oxidative stress model experiments because it can be superheated in an aqueous solution to decompose and form relatively stable peroxyl radicals, triggering free radical chain reactions to generate more species of free radicals, which is closer to what happens in living organisms [25,26,27]. Using antioxidant activity as a tracking index, a sheep erythrocyte oxidation model was constructed to measure the enzymatic activities of catalase (CAT), superoxide dismutase (SOD), glutathione peroxidase (GSH-Px), and the content of malondialdehyde (MDA) to describe the antioxidant activity of FML.

Erythrocyte cell membranes contain a variety of unsaturated fatty acids on their surface, which have a high vulnerability to free radical-mediated oxidative damage and are highly susceptible to lipid peroxidation, causing damage to their cell membrane integrity leading to hemolysis, and some antioxidants such as phytoflavonoids can protect erythrocyte membranes from damage and inhibit hemolysis [28,29,30]. Therefore, the hemolysis inhibition rate of erythrocytes was used to evaluate the protective effect of FML on erythrocytes and its antioxidant activity.

To further explore the antioxidant effect of FML, this study extracted and purified FML and analyzed their physicochemical properties. Furthermore, the research for the first time provides an overview of the antioxidant activity of FML using a hemolysis model of sheep blood erythrocytes. The experimental results provide a research direction for future exploration of FML and provide a theoretical basis for the application and development of FML in the food and pharmaceutical fields.

## 2. Materials and Methods

### 2.1. Materials and Chemicals

Cellulase and pectinase were purchased from Shanghai source leaf Biological Technology Co., Ltd., Shanghai, China. Macroporous resin D101 was purchased from Donghong Chemical Co., Ltd., Xi’an, China. Various flavonoid standards and heparin anticoagulant-containing sheep blood were purchased from Shanghai Yuanye Biotechnology Co., Ltd., Shanghai, China. MS grade organic solvents were employed to run the instrument; the remaining reagents used were analytical grade. 2,2 0-azobis(2-amidinopropane) dihydrochloride (AAPH) was obtained from Shanghai Maclean Biochemical Technology Co., Ltd., Shanghai, China. Phosphate-buffered saline (PBS, pH 7.4) was obtained from Hyclone, USA. Kits to determine the activity of DPPH, ABTS, hydroxyl radicals, superoxide anion generation, SOD, MDA, GSH-PX, CAT, and bicinchoninic acid (BCA) were purchased from Nanjing Jiancheng Institute of Biotechnology, China.

### 2.2. Isolation and Purification of FML

**Figure 1:**
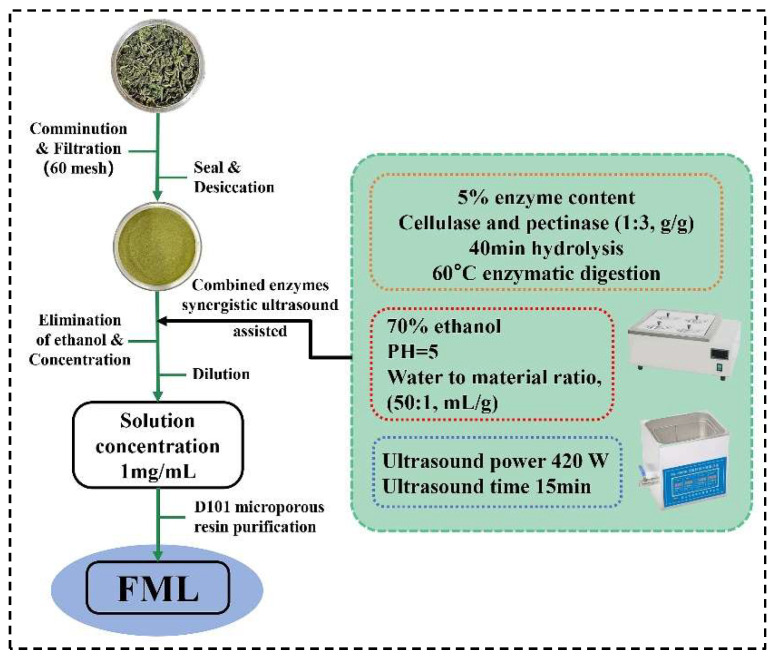
The procedure for the extraction and purification of FML.

Mulberry leaves were obtained from Shanxi, China. The dried plants were ground and filtered through a 60-mesh screen to obtain the homogeneous sample powder, and placed in a desiccator for use. Using the ultrasonic-assisted enzymatic extraction (UAEE), ML was extracted with 70% ethanol of pH 5 (50:1, water to material ratio, mL/g) at 60 °C, 5% cellulase and pectinase (1:3, g/g) was added to hydrolyze for 40 min, and then it was treated with ultrasound (Kunshan Ultrasonic Instruments Co., Ltd., Shanghai, China) at 420 W for 15 min; after filtration, and concentration to remove ethanol, the solution was diluted to 1 mg/mL. The FML were purified by D101 macroporous resin. The extraction and purification process of FML was illustrated in Figure 1.

### 2.3. FML Content Determination

The flavonoid concentration was determined using NaNO_2_ -Al(NO_3_)_3_ -NaOH colorimetric method [24,31] with slight modifications. Briefly, the absorbance was read at 510 nm by a full wavelength enzyme labeler (Burton Instruments Co., Ltd., Gainesville, FL, USA), using rutin as the standard solution and ethanol solution as the blank control. Results were expressed as rutin equivalents (RE) of samples through rutin calibration curve calibration (0.01–0.05 mg/mL) [26]. The FML content and purification were calculated from the content of rutin in the extracts.

Yield (%) = weight of crude FML (g)/weight of ML powder (g)× 100%,(1)

Organ coefficient (%) = (organ weight/body weight) × 100%,(2)

### 2.4. Physicochemical Properties of FML

#### 2.4.1. UV–Visible Spectrum Analysis

The FML and rutin were scanned by ultraviolet in the range of 400–600 nm using a UV-visible spectrum (INESA-L8, INESA Scientific Instrument Co., Ltd., Shanghai, China) and the samples concentrations were 1 mg/mL.

#### 2.4.2. Analysis by Fourier Transform Infrared (FT-IR) Spectroscopy

FML were analyzed using the FT-IR spectrum (PerkinElmer, Waltham, MA, USA). FML (1 mg) were mixed with KBr powder (1:100), ground, and tableted, and then the infrared spectrum was scanned in the frequency range of 4000–500 cm^−1^.

#### 2.4.3. Analysis by LC-MS/MS-Liquid Chromatography with Tandem Mass Spectrometry

After reviewing the relevant literature [16,18,32,33,34,35], 70 polyphenolic standards were selected using a linear concentration gradient, and a single standard was scored to map the ion pairs, followed by qualitative and quantitative analysis of the FML samples.

##### HPLC Conditions

Weigh 0.250 g of the sample in a 15 mL centrifuge tube, add 1.25 mL of 70% methanol solution, shake and mix, soak for 2 h, sonicate for 30 min, centrifuge, take the upper layer of liquid over the membrane in a liquid phase vial to be measured.

The LC-MS/MS employed was a combined system comprising an Agilent HPLC 1100 series and triple quadrupole mass spectrometry API4000. Mobile phase A consists of 0.5% formic acid as the aqueous phase, and mobile phase C consists of acetonitrile as an organic phase used in gradient mode. The initial gradient had 95% mobile phase A, and 5% mobile phase C intended for 8 min. Then, the gradient was changed from 8 min consisting of 75% mobile phase A and 25% mobile phase C held for 4 min. From 12 min, a linear gradient reached 40% mobile phase A as well as 60% mobile phase C, which was held at 1 min. At 13 min, the gradient was shifted to 100% mobile phase B and held for 3.1 min. Soon, the system was turned down to the previous conditions at 16.1 min and held thus for 3.9 min to equilibrate before a fresh injection. The flow rate of the instrument was fixed at 0.6 mL per min. The analytical column used for assays was a 3 × 50 mm Agilent Poroshell 120 EC-C18 column with a particle size of 2.7 µm and column temperature maintained at 35 °C. The instrument was set at 10 μL for sample injection.

##### MS/MS Conditions

The mass spectrometry detection was performed in multiple reaction monitoring (MRM) detection conditions; heated electrospray ionization in negative ion detection mode (ESI-), over the m/z range of 100–1000 Da.; spray voltage 4.5 kv; desolventizing temperature 500 °C; desolventizing gas (N_2_) 1000 L/h; heated electrospray ionization in positive ion detection mode (ESI+), m/z range of 100–1000 Da.; spray voltage 5.5 kv; desolventizing temperature 500 °C; desolventizing gas (N_2_) 1000 L/h.

#### 2.4.4. Analysis by Nuclear Magnetic Resonance Spectroscopy (NMR) 

FML were taken and dissolved in deuterated DMSO and then placed in an NMR tube. The one-dimensional NMR hydrogen (1H) and carbon (13C) spectra in the tubes were examined using a 500 M NMR-171 resonance spectrometer at 25 °C.

### 2.5. Antioxidant Activity of Mycelial Polysaccharides In Vitro

#### 2.5.1. DPPH Radical Scavenging Activity

The DPPH radical scavenging ability kit was used to determine the DPPH· scavenging activity of FML. In brief, the purified FML samples were weighed precisely and configured with distilled water to various concentrations (0.02, 0.04, 0.06, 0.1, 0.2, 0.4, 0.6, 0.8, 1.0, 1.5 and 2.0 mg/mL), while Vc was used as a positive control at the same concentrations. All tests were performed in triplicate. DPPH· scavenging activity was calculated as follows:

DPPH· scavenging activity (%) = [1 − (A_1_ − A_0_)/A_2_] × 100%, (3)

where A_0_ is the absorbance of the sample only (sample without DPPH· solution), A_1_ is the absorbance of the sample with DPPH· solution, and A_2_ is the absorbance of the control (DPPH· solution without sample).

#### 2.5.2. Hydroxyl Radical Scavenging Activity

The hydroxyl radical scavenging rate of FML was determined using a hydroxyl radical assay kit, and Vc was used as the positive control. Briefly, the purified FML and Vc standard were weighed precisely and configured with distilled water to various concentrations (0.0625, 0.125, 0.25, 0.5, 1.0, 2.0, and 5.0 mg/mL), and hydroxyl radical scavenging activity was calculated according to the following formula:

Inhibition rate (%) = [1 − (A_1_ − A_0_)/A_2_] × 100%, (4)

where A_0_ is the absorbance of the sample only (sample without HO· solution), A_1_ is the absorbance of the sample with HO· solution, and A_2_ is the absorbance of the control (HO· solution without sample).

#### 2.5.3. Superoxide Anion Radical Scavenging Activity

The hydroxyl radical scavenging rate of FML was determined using a superoxide anion radical assay kit, and Vc was used as the positive control. Briefly, the purified FML and Vc standard were weighed precisely and configured with distilled water to various concentrations (0.0625, 0.125, 0.25, 0.5, 1.0, 2.0, 3.0, 4.0, and 5.0 mg/mL), and hydroxyl radical scavenging activity was calculated according to the following Equation:

Scavenging ability (%) = (A_0_ − A_1_)/A_0_ × 100%, (5)

where A_0_ was the absorbance value of the control group, and A_1_ was the absorbance value of the sample group.

#### 2.5.4. ABTS Radical Scavenging Activity

The ABTS radical scavenging activity of FML was measured based on the reported method [24,36], slightly modified as follows. The ABTS free radical stock solution was obtained by mixing 7.0 mmol/mL of ABTS solution and 2.45 mmol/mL of K_2_(SO_4_)_2_ solution in equal proportions at room temperature and protected from light for 16 h. The ABTS radical stock solution was diluted with phosphate buffer (10 mmol/L, pH = 7.4) so that the absorbance of the solution was measured between 0.7 ± 0.02 at 734 nm on a full-wavelength enzyme standardization instrument, stored away from light, and set aside. Sample solutions of different concentrations (0.01, 0.015625, 0.03125, 0.0625, 0.125, 0.25, 0.5, 1.0 and 2.0 mg/mL) as well as Vc control sample solutions were prepared using pure water as solvent.

The method was as follows: Aspirate 1.0 mL of the sample solution, add 3.0 mL of diluted ABTS radical stock solution, react at room temperature for 1 h, and measure the absorbance at 734 nm on a full-wavelength enzyme calibrator as A_1_. Take 3.0 mL of K_2_(SO_4_)_2_ solution instead of ABTS-radical stock solution, and record the absorbance as A_2_. Take 1.0 mL of pure water instead of the sample, and record the absorbance as A_3_. The absorbance was recorded as A_3_. The ABTS free radical scavenging activity was calculated as follows:

Scavenging ability (%) = (A_3_ − A_1_ + A_2_)/A_3_ × 100%, (6)

where the absorbance of A_1,_ A_2,_ and A_3_ was as mentioned above.

### 2.6. Preparation of Erythrocyte Suspensions

The AAPH free radical scavenging activities of the flavonoid (FML) samples were slightly modified according to the previously reported method [27,37,38,39]. The AAPH radical scavenging activity of FML was assessed by observing the inhibition of sheep erythrocyte hemolysis. The erythrocytes were isolated by centrifugation (1200× *g*, 10 min, 4 °C) washed three times with PBS (pH 7.4), until erythrocyte precipitation was clear and transparent in the supernatant, and resuspended in the same buffer at a hematocrit concentration of 20%.

### 2.7. AAPH-Treated Erythrocyte Hemolysis Assay

**Figure 2:**
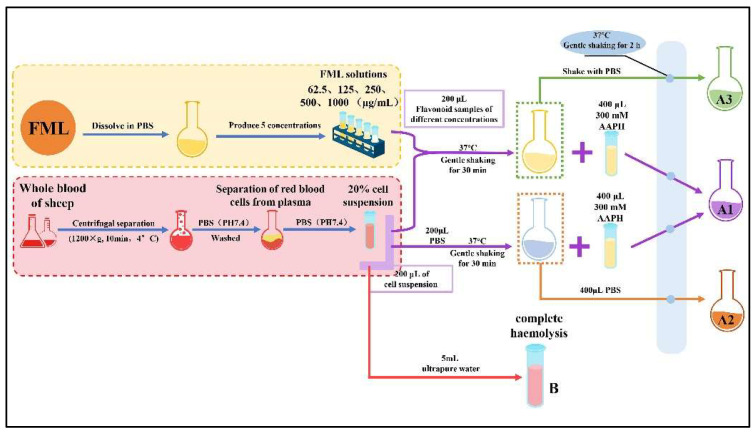
The procedure for the AAPH-treated erythrocyte hemolysis assay.

Specifically, the FML samples were configured with PBS to various concentrations (62.5, 125, 250, 500 and 1000 mg/mL). An aliquot of 200 μL of a 20% erythrocyte suspension was mixed with 200 μL of FML (absorbance A_1_) at different concentrations or PBS (absorbance A_2_). The mixture was incubated at 37 °C for 30 min with gentle shaking, after which 400 μL of 300 mM AAPH was added, and the incubation continued at the same temperature for another 2 h (where the final concentration of AAPH was 150 mM). An amount of 200 μL of different concentrations of FML samples was added to the erythrocyte suspension and co-incubated with PBS as a toxicity control group (A_3_), at the same concentration. Finally, the reaction solution was diluted with 5 mL of PBS (PH 7.4) and centrifuged at 1200× *g* for 10 min at 4 °C, and the absorbance (A_x_) of the supernatant was measured at 540 nm. Complete hemolysis was obtained by adding 5 mL of ultrapure water to the erythrocyte suspension and the absorbance (B) was measured under the same conditions. The procedure for the AAPH-treated erythrocyte hemolysis experiment is shown in Figure 2. The percentage hemolysis was calculated as follows:

hemolysis inhibition (%) = (A_x_/B) × 100%, (7)

where A_x_ refers to the absorbance A_1_, A_2_, A_3,_ and B, as mentioned above.

### 2.8. Investigation of the Antioxidant Ability

Blood cells were collected and washed three times with PBS (pH 7.4), lysed by adding approximately 4 times the volume of ultrapure water, centrifuged (1200× *g*, 10 min, 4 °C) after 10 min of an ice bath, and cell lysis buffer was obtained, then stored at −80 °C before determination. A BCA protein assay kit and a microscale MDA kit were used to monitor the protein and MDA contents, respectively. The Cellular Glutathione Peroxidase Kit, Total Superoxide Dismutase Assay Kit, and Catalase Assay Kit were used to measure the activities of GPx, SOD, and CAT according to the manufacturer’s instructions.

### 2.9. Scanning Electron Microscope (SEM) Analysis

Erythrocyte imaging was carried out using a scanning electron microscope (Mira, Tescan, Czech Republic). Erythrocytes were fixed with moderate amounts of 2.5% glutaraldehyde for 2 h at 4 °C after the pretreatment. Next, a film consisting of a single layer of blood cells was made by spreading the treated blood samples onto newly split mica and washed three times with PBS (pH 7.4). After dehydrating the cells with successive washes of ethanol in ascending concentrations (50%, 70%, 80%, 90%, 95%, and 100%) for 10 min each, the cell samples were coated with gold-palladium, after which the film was placed on the specimen holder and images of the blood samples were acquired.

### 2.10. Statistical Analysis

Experiments were carried out in triplicate, and all data are reported as the mean ± standard deviation (SD). All graphs were created, and calculations were performed using the statistical software (SPSS17.0, Chicago, IL, USA) and GraphPad (Prism 8, San Diego, CA, USA), respectively.

## 3. Results

### 3.1. Isolation and Purification of FML

As shown in Table 1, after ultrasonic-assisted enzymatic extraction (UAEE) extraction, the FML were isolated from ML with a yield of 39.33 ± 0.57 mg/g (dry weight), and the yield was 80.74% after purification by D101 macroporous resin.

### 3.2. Physicochemical Property Analysis

**Figure 3:**
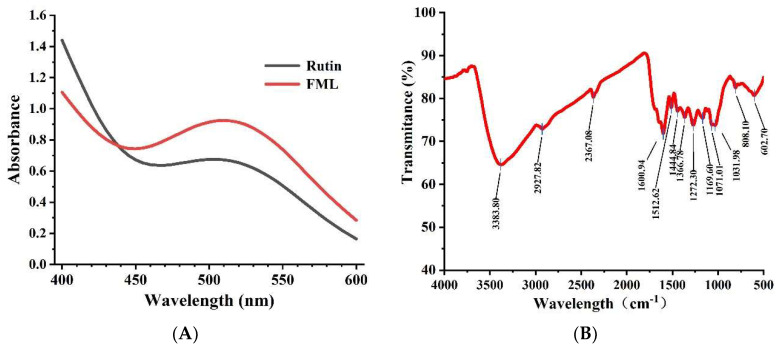
(**A**) UV scan spectrum of FML and rutin; (**B**) FT-IR spectrum of FML.

The UV spectra of FML and rutin at 400–600 nm are shown in Figure 3A. The absorption peak of FML almost coincided with the UV spectrum of rutin, and there was a strong absorption peak at 510 nm, which is the characteristic absorption peak of flavonoids. 

Flavonoids contain hydroxyl, phenolic hydroxyl, methoxy, alkoxy, benzene, and other groups on the parent nucleus and have characteristic IR absorption peaks at 3100–3460 cm^−1^, 1600–1640 cm^−1^, and 1380 cm^−1^, 1260 cm^−1^, and 1090 cm^−1^. FT-IR spectroscopy was used to analyze functional groups of flavonoids. The representative absorption peaks are shown in Figure 3B. The broad and strong absorption peak near 3383 cm^−1^ was attributed to the presence of a large number of phenolic hydroxyl groups, and the absorption peak near 2927 cm^−1^ of FML was derived from the C–H stretching vibration of CH_3_, C_2_, CH, etc. The absorption peak near 1650–1430 cm^−1^ indicated that it contained a benzene ring. The absorption peak between 1275–1020 cm^−1^ was attributed to the stretching vibration of the -C–O–C- bond. The peak near 1375 cm^−1^ was attributed to the C–H variable angle vibration. The absorption peak between 1275 and 1020 cm^−1^ was attributed to the amide group C=O stretching vibration. 

**Figure 4:**
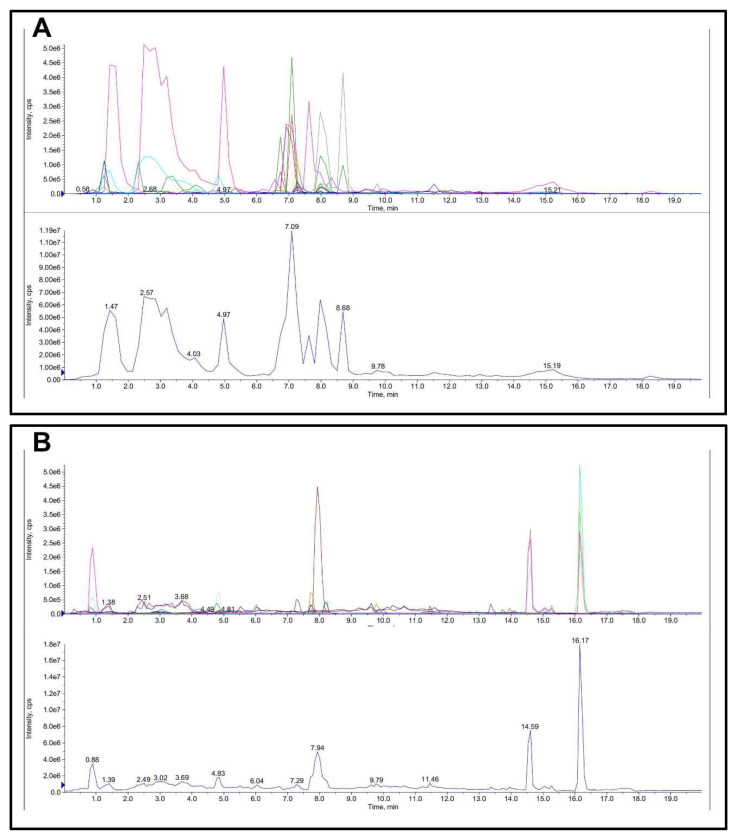
Total ion chromatogram (TIC) of FML in LC-MS/MS: (**A**) ESI-; (**B**) ESI+.

The quantitative and qualitative determination of FML is presented in Table 1 and Figure 4. The main flavonoid compounds in mulberry leaves were hesperidin, rutoside, hyperoside, cyanidin-3-o-glucoside, myricitrin, cyanidin, and quercetin with a content of 10.8, 7.5, 4.098, 3.888, 2.67, 1.17, and 1.116 mg/g FML.

**Figure 5:**
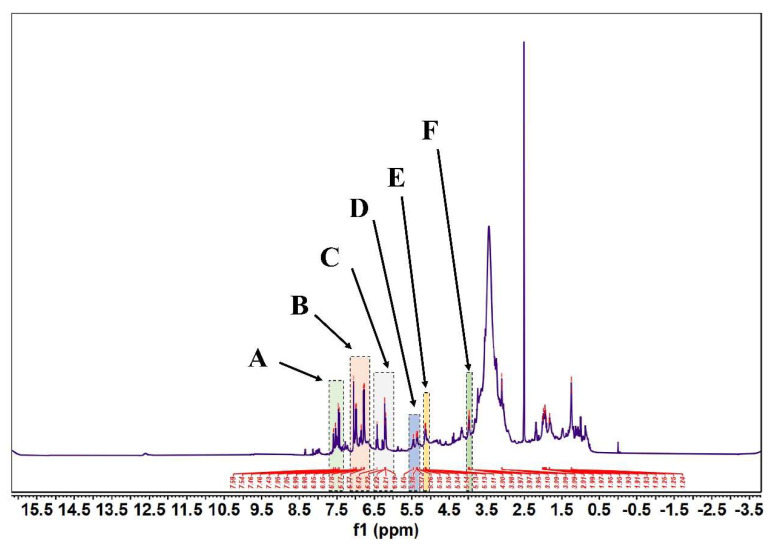
FML NMR hydrogen spectra (1H NMR). (Note: FML had six heterotopic hydrogen signals at δ7.43~7.59(part A), 6.77~7.05(part B), 6.19~6.42(part C), δ5.34~5.45(part D), 5.11~5.14(part E), and 3.95~4.00(part F) ppm in 1H-NMR).

**Figure 6:**
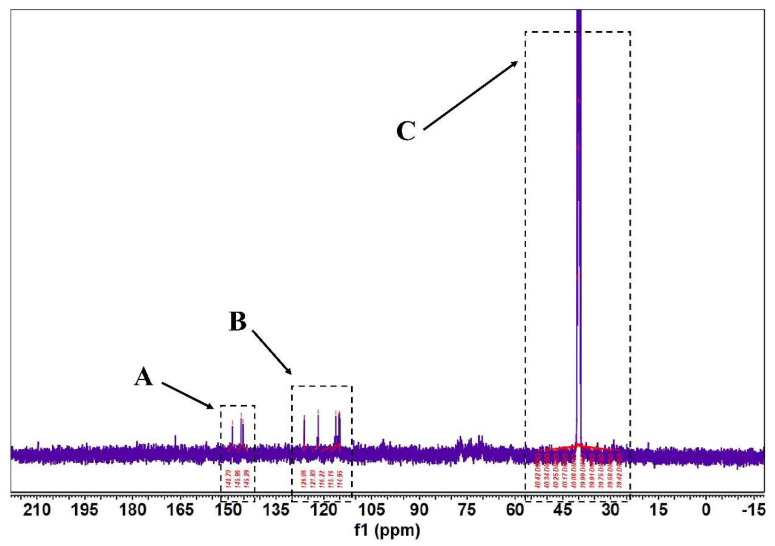
FML NMR carbon spectra (13C NMR). (Note: The δ values of c2~c3 signals in both part A and part B of FML were concentrated between δ 114.95~126.09 and δ 145.29~148.70; Part C is the DMSO–D6).

According to Figure 5 and Figure 6, in the 1H-NMR and 13 C-NMR spectra, δ 6.46~6.09 (m, 2H) were the characteristic proton signals of flavonoids at positions 6 and 8 (when the A-ring 5,7 dihydroxy substituted), while δ H 7.72 and 6.98 were consistent with the proton signals of flavonoids with B-ring para substitution. Based on the physicochemical properties and NMR hydrogen signals we tentatively identified them as flavonoids. The 13C-NMR signals of flavonoids are mostly concentrated in the low-field aromatic signal region. By contrast, the 13C-NMR signals of flavonoid glycosides contained both glycosidic and glycosyl components. According to the signal characteristics of the 13C-NMR spectra of the three carbons in the center of flavonoids, the δ values of C2 were between δ 145.29 and 148.70.0, and the δ values of C3 were between δ 114.95 and 126.09. This chemical signal is characteristic of flavonoids, and the results of 13C-NMR and 1H-NMR spectra are consistent with the analysis of IR for the major flavonoids.

### 3.3. The Antioxidant Activity In Vitro

#### 3.3.1. Ability of FML to Scavenge DPPH

**Figure 7:**
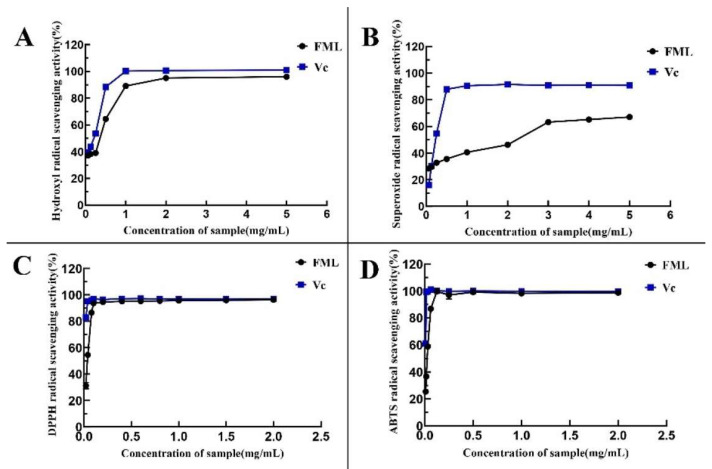
Determination of the antioxidant activity of the five compounds: (**A**) DPPH· scavenging ability; (**B**) ·OH scavenging ability; (**C**) O_2_^−^ scavenging ability; (**D**) ABTS scavenging ability. Vc, vitamin C.

1,1-Diphenyl-2-picrylhydrazyl (DPPH) is one of the stable nitrogen-centered free radicals and a free radical representative to be effectively scavenged by antioxidants19, with strong absorption at 517 nm. The measurement of DPPH· scavenging ability has been widely used to evaluate antioxidant activity. In recent years, flavonoids have been gaining more attention because of their advantages consisting of strong antioxidant activity and few side effects. As shown in Figure 7A, FML showed excellent DPPH· scavenging activity in a dose-dependent manner within the tested concentration range. Specifically, FML were able to scavenge more DPPH· as their concentration increased within the range of 0–0.1 mg/mL. When the concentration was higher than 0.1 mg/mL, the DPPH radical scavenging rate tended to be stable and close to that of Vc, and the scavenging rate of FML on DPPH · was 93.58%, with IC_50_ of 0.0105 mg/mL and 0.0452 mg/mL, respectively, indicating that FML have good scavenging ability for DPPH radicals.

#### 3.3.2. Ability of FML to Scavenge HO·

The hydroxyl radical is considered to be the most reactive of the reactive oxygen species, which could induce damage to adjacent biomolecules, and cause several diseases [15,20,21]. Figure 7B depicts the scavenging ability of FML on HO· scavenging ability compared with Vc as a positive standard, which revealed that FML scavenging occurred in a concentration-dependent manner but it was weaker than that of Vc. The IC_50_ values for the scavenging of hydroxyl radicals were 0.5242 mg/mL and 0.3524 mg/mL, respectively, indicating that FML have a better scavenging ability for hydroxyl radicals.

#### 3.3.3. Superoxide Anion Radical Scavenging Activity

In measurements of the scavenging ability of FML and Vc for superoxide anion, the absorbance reflects the strength of free radical scavenging. As shown in Figure 7C, the scavenging activity of FML for superoxide anion increased slowly in the concentration within the range of 0–5 mg/mL, with radical scavenging activity ranging from 30.26% to 67.06%, and the inhibitory effect of the same concentration was significantly lower than that of Vc (*p* < 0.05), as Vc is a well recognized reducing agent.

#### 3.3.4. ABTS Radical Scavenging Capacity

As shown in Figure 7D, the ABTS radical scavenging rate of FML increased in a concentration-dependent manner in the concentration ranges of 0–0.125 mg/mL. When the concentration was at 0.125 mg/mL, the clearance rate reached 100%, and the IC_50_ for FML and Vc were 0.0319 mg/mL and 0.0082 mg/mL, respectively, suggesting that FML can be considered a good scavenger of ABTS, and would be beneficial to a certain extent in protecting against oxidative damage. The IC_50_ values of antioxidant activity of FML and V_C_ are shown in Table 2.

### 3.4. Effects of FML on AAPH-Induced Erythrocyte Hemolysis

**Figure 8:**
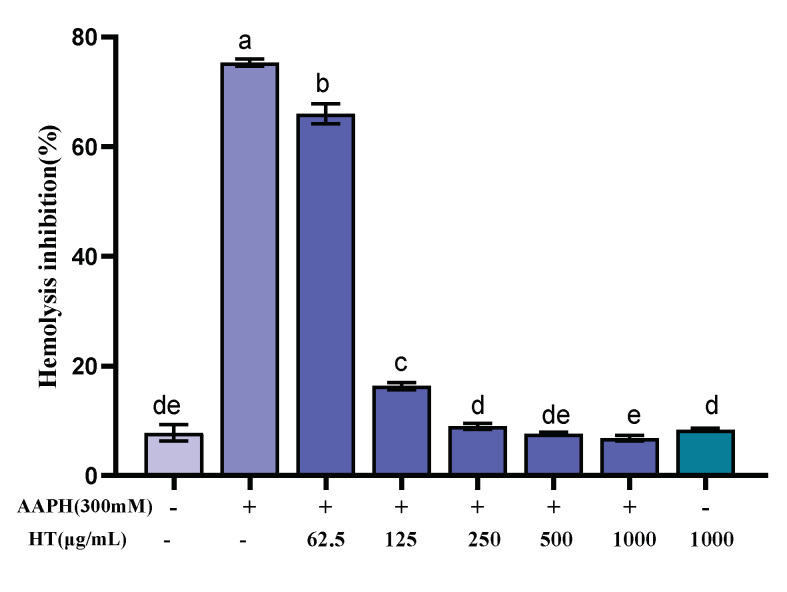
Inhibitory effects of various concentrations of FML on AAPH-induced erythrocyte hemolysis (Note: The same small letters indicate no significant difference between groups, different small letters indicate a significant difference between groups, *p* < 0.05).

Erythrocytes have a sensitive oxidative response and a simple metabolic mechanism, making them an ideal model for studying free radical damage to biological membranes, and thus the erythrocyte hemolysis method was used to study the antioxidant activity of FML. In the erythrocyte hemolysis assay, AAPH, which can decompose at 37 °C to generate an alkyl radical, attacks erythrocyte membrane components and causes changes in the structure and function of cell membranes, leading erythrocyte to hemolysis [25,26,40,41]. As shown in Figure 8, FML exhibited dose-dependent protection on erythrocytes against AAPH-induced hemolysis. The hemolysis rate was decreased as FML concentration increased from 0 to 1000 μg/mL. Under the same incubation conditions, compared with the AAPH experimental group, the hemolysis rate of FML at a dose of 1000 μg/mL, on erythrocyte hemolysis was 6.84%, which was comparable to that of the negative control group with an inhibition rate of 7.81%. When the erythrocytes were treated with FML alone at the highest concentration, the hemolysis rates were similar to those in negative control groups (untreated cells). The results suggested that FML was nontoxic and did not induce erythrocyte damage.

### 3.5. Accumulation of MDA in Erythrocytes after FML Treatment

**Figure 9:**
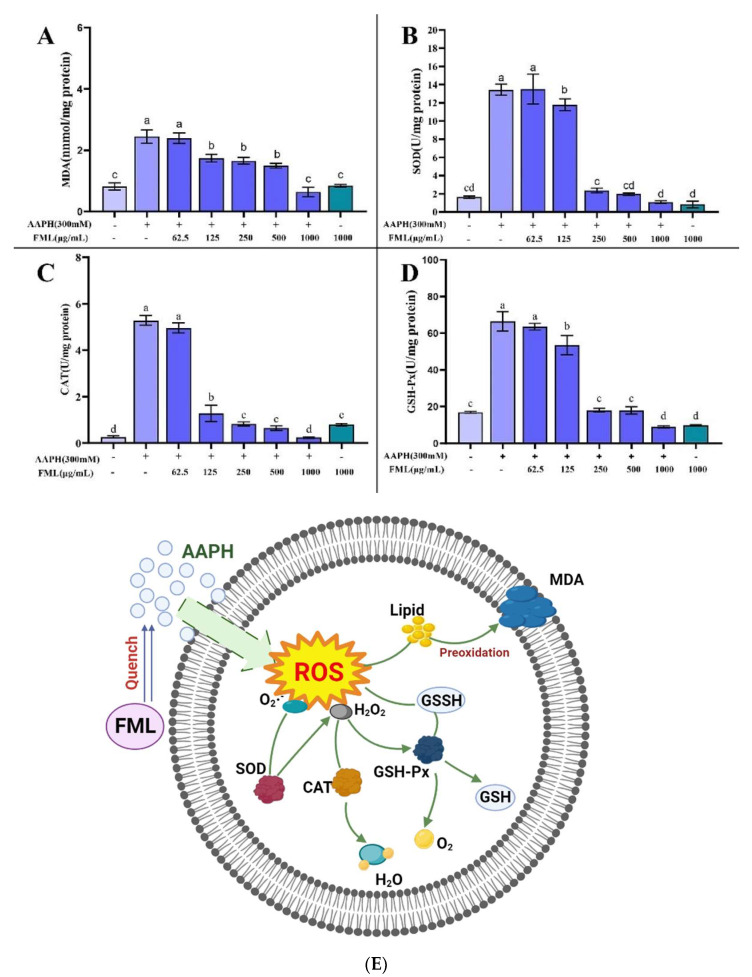
Effect of FML on changes in MDA content (**A**) and enzyme activities of SOD (**B**), GPx (**C**), and CAT (**D**) in erythrocytes with AAPH-induced oxidative damage; (**E**) possible intracellular antioxidant mechanisms of FML in an AAPH-induced model of oxidative damage (Note: The same small letters indicate no significant difference between groups, different small letters indicate a significant difference between groups, *p* < 0.05).

The antioxidant effect of flavonoids is explained by the direct inhibition of AAPH free radicals in erythrocytes, thereby reducing the level of antioxidant-related enzymes and metabolites [27,39]. MDA is one of the important end-products of lipid peroxidation, which affects the structure and function of cell membranes by inducing cross-linking of protein-free amino groups in cell membranes, ultimately causing cytotoxic effects [25,39,42]. Thus, the MDA content is used as an indicator of the degree of lipid peroxidation, from which we can infer that the cell membrane is damaged. FML with an antioxidant effect can inhibit AAPH-induced changes in MDA content as well as antioxidant enzyme activity, which can reduce cell membrane lipid peroxidation and thus decrease the hemolysis rate. Figure 9A shows the effects of FML on the generation of MDA. The AAPH-treated group accumulated significant levels of MDA; the MDA level in erythrocytes was significantly increased from 0.82 to 2.52 nmol/mg protein after treatment with 300 mM AAPH for 2 h, indicating that AAPH induced severe lipid peroxidation in the erythrocytes. However, FML pretreatment significantly decreased the MDA levels of AAPH-treated cells in a dose-dependent manner. Especially at the high FML concentration (250 μg/mL), the MDA concentration declined almost to that of the negative control erythrocytes. For cells incubated with FML without AAPH supplementation, the MDA level was comparable to that of the control, showing that FML themselves do not induce MDA formation. The above results indicated that FML could inhibit the accumulation of MDA, thus reducing the damage to the cell membrane by AAPH.

### 3.6. Changes in CAT, SOD, and GSH-Px Activity in Erythrocytes after FML Treatment

It has been shown that erythrocytes also possess a variety of antioxidant enzymes to defend against intracellular oxidative stress, such as CAT, SOD, and GSH-Px, which play a crucial role in the defense system against oxidative stress [26]. SOD and CAT enzymes are the first line of cellular defense against oxidative injury. SOD can catalyze the conversion of highly reactive superoxide anions into less reactive oxygen species and hydrogen peroxide, and is the primary antioxidant enzyme in the human body [43]. CAT is responsible for the decomposition of H_2_O_2_ into H_2_O and O_2_, protecting cells from the toxicity of H_2_O_2_. GSH-Px decomposes H_2_O_2_ and organic peroxides (ROOH) and is essential for the detoxification of free radicals. Measuring the average enzyme activity of antioxidant enzymes in surviving erythrocytes after AAPH injury revealed that more erythrocytes with low enzyme activity survived because they were protected by the sample, and therefore the average enzyme activity measured was lower.

Figure 9 shows the changes in the activity of SOD, CAT, and GSH-Px after AAPH treatment of erythrocytes. A significant increase in enzyme activity was observed after incubation with AAPH alone, indicating a positive response of the cellular defense system to oxidative stress. The mean enzyme activities of SOD, CAT, and GSH-Px in the surviving erythrocytes of the positive control group that was treated with AAPH were 13.43, 5.26, and 66.53 U/mg protein, respectively, which were higher than the mean enzyme activities of 1.64, 0.26, and 16.88 U/mg protein in the negative control group (without AAPH treatment). In contrast, pretreatment with the addition of different concentrations of FML protected some of the erythrocytes from free radical attack, thus reducing the enzyme activity. The SOD, CAT, and GSH-Px enzyme activities of the experimental group treated with high doses of FML were not significantly different from those of the negative control group (*p* < 0.05).

### 3.7. Effect of FML Treatment on the Erythrocyte Cell Membrane

**Figure 10:**
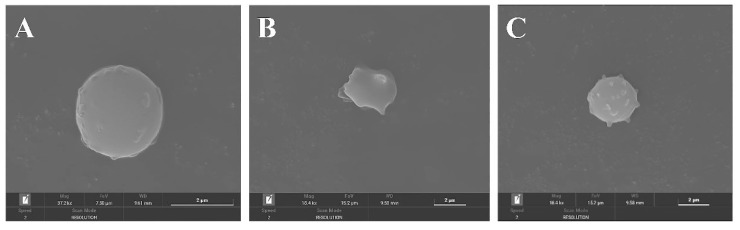
SEM micrographs of sheep erythrocyte samples: (**A**) normal erythrocyte; (**B**) AAPH-treated erythrocyte; (**C**) erythrocyte preincubated with diosmetin before AAPH treatment.

To characterize the erythrocyte damage induced by AAPH and the protection of FML, morphological changes in erythrocytes were examined by SEM. Figure 10A shows the normal amniotic erythrocytes in the negative control group, which are round and have smooth surfaces. Figure 10B shows the positive control group with obvious cell membrane wrinkling, loss of normal biconcave discoid shape of erythrocytes, and a spiny configuration on its surface. However, this damage was dramatically reduced in cells pretreated with 1000 μg/mL diosmetin before the addition of AAPH (Figure 10C), where the cell membrane surface was slightly spiked, but the basic structure of erythrocytes was maintained, and this damage was significantly reduced. In conclusion, the above results confirmed the protective effect of FML on AAPH-induced morphological changes in the red cell.

## 4. Discussion

Flavonoids are natural active substances commonly found in natural plants and are important active ingredients in traditional Chinese medicine with important research significance. Flavonoids have been studied extensively because of their high content in plants, easy availability, and clear pharmacological effects. Previous studies have proved that flavonoids have antioxidant, anti-aging, antibacterial, anti-inflammatory, and other physiological activities, and these physiological activities [44,45] are closely related to their chelating ability with other substances and their antioxidant activity.

The number of free radicals maintains a dynamic balance, and when there is a large accumulation of free radicals in the body, it will cause aging and disease. The damage caused to the body due to oxidative stress can be alleviated through the intake of appropriate amounts of antioxidant substances, and it has been found that antioxidant components can effectively scavenge reactive oxygen radicals and alleviate oxidative stress in the body [46]. Antioxidants can be defined as a class of substances that, at low concentrations, achieve the mitigation, amelioration, or elimination of oxidative damage. The antioxidant mechanism includes complexation with metal ions: free metal ions in the body have strong catalytic properties, which can promote the formation of reactive oxygen radicals; the antioxidant activity of flavonoids is closely related to their structural relationship, and some functional groups in flavonoids can complex with metal ions to achieve the effect of scavenging excess metal ions in the body [47]. There is also an effect on the body enzymes: some oxidases can promote the formation of free radicals, and flavonoids can react with oxidase to reduce the formation of free radicals, and terminate the free radical chain reaction; the phenolic hydroxyl group in flavonoids can react with free radicals to generate a stable semi-quinone structure, terminating the reaction of the free radical chain [48,49].

Due to the increasing demand for quality of life and the hidden dangers of synthetic antioxidant use, the clear efficacy and safety of natural antioxidants means that they will gradually replace chemical antioxidants, so it is promising to search for antioxidants from natural plants and study their antioxidant mechanisms and synergistic antioxidant effects. ML has good application prospects as a medicinal food drug. FML have more biological activities, including antibacterial, antioxidant, analgesic, anti-inflammatory, hypoglycemic, and hypolipidemic activities. [8,14,15,20,36,50,51,52]. They are biological active ingredients with great research significance, which can be developed into food and functional health products. There is a trend of extracting active ingredients from traditional Chinese medicine for the development of food and functional health products.

The UV and IR spectra proved that FML have the characteristic peaks of flavonoids, indicating that FML are typical flavonoids. In the in vitro antioxidant assay, FML had noticeable scavenging activities on 2,2-diphenyl-1-picryl-hydrazyl (DPPH) radical, hydroxyl radical, superoxide anion, and ABTS radical with IC_50_ values of 0.0452 mg/mL, 0.5242 mg/mL, 3.1768 mg/mL, and 0.0319 mg/mL. In the AAPH-induced erythrocyte hemolysis model, the hemolysis rate of the experimental group was 6.84% after high FML treatment, which was significantly lower than that of the AAPH group at 75.33%. The enzymatic activities of SOD, CAT, and GSH-Px were 1.08, 0.24, and 8.96 U/mg protein, respectively, which were lower than those of the AAPH-treated group; in the non-enzymatic antioxidant system, the level of malondialdehyde (MDA) was 0.64 nmol/mg protein after high FML treatment. In addition, both SEM and MDA showed that FML exhibited excellent intracellular antioxidant activity on erythrocytes and attenuated AAPH-induced erythrocyte hemolysis, maintaining normal levels of active antioxidant enzymes in the cells. Utilizing the antioxidant properties of FML is expected to provide a theoretical basis for the use of ML as a good source of natural antioxidants, antioxidant compounds, and functional food antioxidants.

Oxidative reactions are the basis of the organism’s life activity, and in living organisms, the mitochondrial oxidative respiration electron transport chain is the most common pathway for the generation of free radicals [53]. There are many types of free radicals, and most studies focus on oxygen (O) and nitrogen (N) as the central reactive groups, among which ROS radicals are the most studied. Moderate ROS radicals have antibacterial, vasodilator, signal transduction, and other physiological effects on the body [54]. In addition, the antioxidant system in the body eliminates excess ROS radicals and keeps the internal environment in a stable state. However, excessive ROS radicals in the body can induce oxidative damage to cells, damaging proteins, DNA, and other macromolecules, thus causing a series of diseases related to oxidative damage [55]. Although many chemicals such as V_C_ are able to reduce oxidative stress, natural antioxidants are still a hot topic of research. As a natural antioxidant, flavonoids are mainly used to scavenge free radicals, enhance reducing power, and inhibit lipid peroxidation through electron transfer or as hydrogen donors, and their ability to scavenge free radicals is closely related to their pharmacological activity [56]. Yamakoshi et al. [57] found that flavonoids in grape seed extract can chelate metal ions, scavenge free radicals, and act as inhibitors of lipid peroxidation reactions. Alessia Remigante et al. [28] demonstrated that Açai extract prevented D-Gal-induced OS damage, including ROS production, lipid peroxidation, and total protein sulfhydryl oxidation, and restored the distribution of B3p and CD47 on the plasma membrane.

The results of this experiment showed that FML could inhibit the hemolysis and MDA content of sheep erythrocytes induced by AAPH, and the MDA content of erythrocytes in sheep erythrocytes increased significantly after 2 h of AAPH treatment; and FML could significantly reduce the oxidative hemolysis rate and the oxidation product MDA content of erythrocytes within the sample concentration range. The SEM micrographs again demonstrated that the basic structure of erythrocytes was maintained after FML pretreatment, probably because FML scavenged the free radicals generated by AAPH, thereby stabilizing the phospholipid bilayer structure of the cell membrane, protecting the cell membrane, and enhancing the tolerance of the cell to free radicals.

## 5. Conclusions

A large body of evidence shows that aging is associated with the degradation of antioxidant status. In this study, FML were extracted using ultrasonic-assisted enzymatic extraction (UAEE) and purified by D101 macroporous resin. Through LC-MS/MS, UV, IR, and NMR analysis, the types of FML were identified. In vitro, the antioxidant activity assay showed that FML exhibited significant scavenging activity against DPPH radical, hydroxyl radical, superoxide anion radical, and ABTS radical. The protective effect of FML against AAPH-induced oxidative stress was investigated by using erythrocytes with AAPH-induced damage as a model. The results showed that FML could protect cell membranes from oxidative damage by free radicals by inhibiting AAPH-induced oxidative damage in erythrocytes and reducing MDA enzyme activity, which reduced the hemolysis rate to some extent. This experiment provides a theoretical basis that mulberry leaves can be used as a functional food with good application prospects.

## Figures and Tables

**Table 1:** Multiple reaction monitoring (MRM) details for liquid chromatography with tandem mass spectrometry (LC-MS/MS) estimation of FML from the analytical data of a biological replicate.

	Compound	Formula (M)	Ion Mode	MW (g/mol)	Quantitative Ion Pairs (Da)	Retention Time (min)	Calculated Conc. (ng/mL)
Rutoside		C_27_H_30_O_16_	ESI-	610.52	609.300/300.100	7.09	125,000
Hyperoside		C_21_H_20_O_12_	ESI-	464.38	462.837/254.940	9.37	68,300
Catechin		C_15_H_14_O_6_	ESI-	290.2681	289.100/245.000	5.32	163
Myricitrin		C_21_H_20_O_12_	ESI-	464.38	462.901/270.937	7.27	44,500
Isoquercitrin		C_21_H_20_O_12_	ESI-	448.38	463.000/254.900	9.34	43.1
Kaempferol 3- rutinoside		C_27_H_30_O_15_	ESI-	594.518	592.914/254.911	7.98	15,700
Paeoniflorin		C_23_H_28_O_11_	ESI-	480.47	479.100/120.900	5.90	3570
Epicatechin		C_15_H_14_O_6_	ESI-	290.27	289.100/109.000	4.97	64.2
Taxifolin		C_15_H_12_O_7_	ESI-	304.25	303.100/124.900	6.85	518
Quercetin		C_15_H_10_O_7_	ESI-	302.24	301.000/150.900	10.1	886
Luteolin		C_15_H_10_O_6_	ESI-	286.23	285.100/132.900	10.1	95.6
Morin		C_15_H_10_O_7_	ESI-	302.24	300.825/150.986	10.1	698
Astragalin		C_21_H_20_O_11_	ESI-	286.25	446.901/226.984	8.06	16,700
Quercitrin		C_21_H_20_O_11_	ESI-	448.38	446.901/254.942	8.03	18,600
Isorhamnetin-3-O-glucoside		C_22_H_22_O_12_	ESI-	478.4	477.100/313.900	8.33	3.65
Curculigoside		C_22_H_26_O_11_	ESI-	466.44	465.100/136.900	8.33	23.0
Hesperidin		C_28_H_34_O_15_	ESI-	610.56	609.300/301.100	6.97	180,000
Cynaroside		C_21_H_20_O_11_	ESI-	448.37	446.891/284.780	7.99	16,200
Vitexin		C_21_H_20_O_10_	ESI-	432.38	431.100/310.900	8.21	4990
Kaempferol		C_15_H_10_O_6_	ESI-	756.66	285.100/185.100	11.2	209
Guaiaverin		C_20_H_18_O_11_	ESI-	434.35	432.871/299.937	7.45	12,300
Licochalcone-A		C_21_H_22_O_4_	ESI-	338.4	337.300/120.000	13.8	15.2
Ginsenoside Rg2		C_42_H_72_O_13_	ESI-	785.03	783.500/475.200	12.1	116
Ginsenoside C-K		C_36_H_62_O_8_	ESI-	622.88	621.500/161.000	14.2	211
Ginsenoside Rc		C_53_H_90_O_22_	ESI-	1079.27	1077.600/945.700	12.0	230
Ginsenoside Rg1		C_42_H_72_O_14_	ESI-	801.01	799.500/475.300	11.5	1150
Ginsenoside Rf		C_42_H_72_O_14_	ESI-	801.02	799.500/475.250	11.4	4.54
Ginsenoside F1		C_36_H_62_O_9_	ESI-	638.87	637.600/475.300	12.0	3140
Ginsenoside Rb2		C_53_H_90_O_22_	ESI-	1079.27	1077.600/783.500	11.5	110
Ginsenoside Rd		C_48_H_82_O_18_	ESI-	947.15	945.600/621.500	12.1	71.2
Ginsenoside Re		C_48_H_82_O_18_	ESI-	947.17	946.000/783.500	12.2	284
Hesperetin		C_16_H_14_O_6_	ESI-	302.28	301.100/150.900	9.94	1190
Polydatin		C_20_H_22_O_8_	ESI-	390.38	391.100/229.100	6.63	318
Cyanidin 3-O-glucoside		C_21_H_21_ClO_11_	ESI+	484.84	448.861/286.932	7.94	64,800
Cyanidin 3-O-rutinoside		C_27_H_31_O_15_	ESI+	594	594.874/286.961	7.74	39,600
Isorhamnetin-3-O-glucoside		C_22_H_22_O_12_	ESI+	624.54	624.870/316.943	8.01	191
Naringenin		C_15_H_12_O_5_	ESI+	272	272.968/147.102	10.4	341
Apigenin 7-glucoside		C_21_H_20_O_10_	ESI+	432.38	433.200/271.100	8.19	6680
Cyanidin		C_21_H_28_O_8_	ESI+	322.7	286.900/109.100	11.1	19,500

**Table 2:** IC_50_ values of FML and Vc in antioxidant activity.

Samples	IC_50_(mg/mL)			
	DPPH	·OH	O_2_^−^	ABTS
Vc	0.0105	0.3524	0.2355	0.0082
FML	0.0452	0.5242	3.1768	0.0319

## Data Availability

Not applicable.

## Sample Availability

Samples of the compounds are not available from the authors.
